# Macronutrient intakes and cardio metabolic risk factors in high BMI African American children

**DOI:** 10.1186/1743-7075-6-41

**Published:** 2009-10-13

**Authors:** Sushma Sharma, Lindsay S Roberts, Mark L Hudes, Robert H Lustig, Sharon E Fleming

**Affiliations:** 1 The Dr Robert C and Veronica Atkins Center for Weight and Health, University of California, Berkeley, CA 94720-3100, USA; 2 Department of Nutritional Sciences and Toxicology, University of California, Berkeley, CA 94720-3104, USA; 3 Division of Pediatric Endocrinology, University of California, San Francisco, CA 94143, USA

## Abstract

**Background:**

The aim of this study was to evaluate the relationship between intakes of energy-providing macronutrients, and markers of cardio metabolic risk factors in high BMI African American (AA) children.

**Methods:**

A cross sectional analysis of a sample of 9-11 year old children (n = 80) with BMI greater then the 85^th ^percentile. Fasting hematological and biochemical measurements, and blood pressure were measured as selected markers of cardio metabolic risk factors and their relationships to dietary intakes determined.

**Results:**

After adjusting for gender, pubertal stage and waist circumference (WC), multivariate regression analysis showed that higher total energy intakes (when unadjusted for source of energy) were associated with higher plasma concentrations of intermediate density lipoprotein cholesterol (IDL-C) and very low density lipoprotein cholesterol (VLDL-C). Higher intakes of carbohydrate energy (fat and protein held constant) were associated with higher IDL-C, VLDL-C, triglycerides (TG) and homeostasis model assessment of insulin resistance (HOMA-IR). Higher intakes of fat (carbohydrate and protein held constant), however, were associated with lower IDL-C; and higher protein intakes (fat and carbohydrate held constant) were associated with lower HOMA-IR.

**Conclusion:**

The specific macronutrients that contribute energy are significantly associated with a wide range of cardio metabolic risk factors in high BMI AA children. Increases in carbohydrate energy were associated with undesirable effects including increases in several classes of plasma lipids and HOMA-IR. Increases in protein energy were associated with the desirable effect of reduced HOMA-IR, and fat energy intakes were associated with the desirable effect of reduced IDL-C. This analysis suggests that the effect of increased energy on risk of developing cardio metabolic risk factors is influenced by the source of that energy.

## Background

In the past three decades, the prevalence of overweight and obesity among youth in the USA has doubled and tripled, respectively [1]. Obesity during childhood and adolescence is associated with a number of cardiovascular disease risk factors including type 2 diabetes mellitus, hypertension, and dyslipidemia. Many studies including the Muscatine Study [2,3] and the Bogalusa Heart Study [4] have convincingly shown that overweight and obesity during adolescence is a determinant of a number of CVD risk factors in adulthood. A clinical report published by the American Academy of Pediatrics also suggests that because of their high risk of developing CVD, overweight children are in need of cholesterol screening regardless of family history or other risk factors [5].

Ethnicity also plays an important role in the development of the cardio metabolic syndrome. The African American (AA) population has been shown to be at higher risk for the metabolic syndrome than whites [6] and, as the prevalence of obesity and the metabolic syndrome in AA children is on the rise [7], risk factors management should begin at an early age.

Recently, there has been growing evidence that childhood diet may have important implications for the development of obesity and chronic disease in later life. Health professionals have suggested that the dietary habits of children and adolescents need serious attention. It has been established that management of energy intakes from different macronutrients plays a more important role in the development of obesity and metabolic complications than does total energy alone. Studies examining the early natural history of heart disease have demonstrated the relationship between early dietary habits, such as high intake of energy from fat, and the subsequent development of CVD [8,9]. Replacement of dietary saturated fat with carbohydrate has been shown to increase triglycerides (TG) in adults [10]. Similarly, studies have found an association between protein intake in childhood and changes in body fatness later in life, generating a hypothesis that high levels of protein consumption in early childhood may lead to the later development of obesity [11,12]. Thus, metabolic diseases in later life may be attenuated by careful selection of which macronutrients are consumed during childhood, and the amount of each.

Efforts have been made to investigate the influence of dietary components on selected cardio metabolic risk factors in adults [13] and in children of various ethnicities [14-16]. Most studies, however, have studied replacement effects where, by definition, intakes of at least 2 energy-providing dietary components are altered. Such an approach cannot determine which component of these components is responsible for observed effects. Thus, the main objective of this study was to determine the relationship between each dietary macronutrient independently of the other two, and cardio metabolic risk factors in high BMI AA children.

## Methods

### Subjects

Subjects included in this analysis represented a cross-sectional convenience sample of children enrolled in *Taking Action Together*, an ongoing 2-year YMCA-based intervention trial to reduce the risk of diabetes in high BMI 9-11 year old African American children from inner-city urban neighborhoods. All participants were 9-11 years old, with high BMI's above the 85th percentile, fasting glucose <120 mg/dl, free from any known metabolic diseases, and were not taking medications known to affect the study outcomes. Study participants were recruited by distributing pamphlets at local recreational sites and schools in inner-city Oakland, CA. Parental informed consent were obtained for all subjects, and all protocols were approved by institutional review boards at the University of California, Berkeley and San Francisco. A complete set of data were available for a total 84 children (38 boys and 46 girls).

### Anthropometric Measurements

Body weight and height were measured to the nearest 0.1 kg and 0.1 cm using a digital electronic scale (BWB 800, Tanita, Japan), and a portable stadiometer, respectively. Body mass index (BMI), BMI percentiles and BMI z-scores were generated using an age and gender specific CDC calculator . For simplicity, the term "high BMI" has been used in this paper to collectively refer to children defined by an expert committees, and based on CDC growth charts [17-19], as being either "at risk of overweight". (BMI's between 85th and 95th percentil) or "Overweight/Obese" (BMI's at or above 95th percentile).

Using a plastic non-elastic measuring tape, waist circumference was measured just above the iliac crest with child the standing position. Measurements were taken twice and if agreement between repeats was greater than 0.4 cm, a third measurement was taken and the mean calculated using the closest two values [20]. Total body resistance was measured using bioelectrical impedance analysis (Model BIA-101Q; RJL systems, Detroit, MI) according to manufacturer's instructions [20], and percent body fat was computed using Horlick's equation [21]. This method and equation have been validated in AA children previously.

### Blood Pressure (BP) Measurements

Blood pressure measurements were taken between 0800 and 1200 hr. Children were asked to rest for a duration of 15-20 minutes in a sitting position after which they were measured with a manual sphygmomanometer by highly trained individuals. Measurements were repeated until two consecutive systolic and diastolic measurements agreed within 4 and 2 mm Hg, respectively. This series of measurements was repeated twice in children, with repeats spaced at least 2 hr apart, and the second series of measurements used for analyses. Data were analyzed using both systolic and diastolic pressures, and after conversion to blood pressure percentiles (matched for age, height and gender) using regression equations developed and reported elsewhere [22].

### Biochemical Measurements

Subjects reported to Children's Hospital and Research Center, Oakland CA after a 12 hr overnight fasting when their blood was drawn. Plasma lipids were measured by a comprehensive lipoprotein analysis performed by a commercial lab (LabCorp). Using the vertical auto profile (VAP)-cholesterol method a modified density gradient centrifugation technique, concentrations of the cholesterol content of all lipoprotein subclasses (total cholesterol, HDL-cholesterol, LDL-cholesterol, IDL-cholesterol, VLDL-cholesterol) and triglycerides were determined on a single sample [23].

Fasting plasma glucose was determined using a hexokinase- peroxidase method (glucose HK-60 assay; Diagnostic Chemicals, Oxford, CT). Reference standards of normal and elevated glucose concentration (DC-Trol;Diagnostic Chemicals, Oxford, CT) were analysed with each assay for quality-control purposes.

Fasting serum insulin concentrations were determined using enzyme-immunoassay (Linco, St. Charles, MO). The insulin assay has <1% cross-reactivity with proinsulin. The intra-assay variation was 4.5% and inter-assay variation was 10%

Fasting glucose and insulin values were used to calculate insulin resistance [24] using the homeostasis model assessment of insulin resistance (HOMA-IR), defined as fasting glucose (mmol/l) × insulin (μU/ml)/22.5.

Serum non esterified free fatty acids (NEFA) were measured using an in vitro enzymatic colorimetric assay (NEFA, Wako Diagnostics, USA) and used to calculate Fatty acid insulin sensitivity (ISI-FFA) by the formula:{2/(insulin × NEFA) +1 } [25].

Pubertal development was assessed by measurements of serum luteinizing hormones (LH) in boys and estradiol and LH in girls. Children were classified into stages 1 through 5 using literature-derived values [26-31]. The hormone concentrations included in the 5-pubertal stages were as follows: 0-11.6, 11.61-26.2; 26.21-50.7; 50.71-64.9 and >64.91 pg/mL for E_2 _in girls; 0-0.4, 0.41-3.4; 3.41-4.1; 4.11-5.9 and >5.91 IU/L for LH in girls; and 0-0.7, 0.71-1.6; 1.61-2.6; 2.61-3.1 and >3.11 IU/L for LH in boys. When E_2 _and LH hormone concentrations in girls suggested different pubertal stages, the higher stage was assigned to that girl [31].

### Energy intake

Three-day food diaries were used to assess intakes of nutrients as they have been shown to be a more accurate tool for diet assessment than 24-h diet recall or food frequency questionnaire methods [32]. Before completing a 3-day food diary (2 weekdays and 1 weekend day), participating children and a parent/guardian were trained to record portion sizes, brand names, place consumed, and time of day food was consumed. Trained staff reviewed the questionnaires with the child, expanding on descriptions of foods and portion sizes when needed.

Macronutrient intakes were determined using the USDA nutrient database [33]. To do this, the foods listed on the 3-day food diaries were labeled according to the 8-digit food codes, portion sizes or weights were entered into this software, and computer programs were used to calculate the 3-day average intakes of energy and specific macronutrients. Analyses were carried out on the data set as a whole and, following the protocol for the National Health and Nutrition Examination Survey (NHANES), no quantification or exclusion for underreporting or over reporting was made. Average daily intakes of energy (kcal/day) and of the macronutrients, carbohydrates, fat and protein (g/day) were used for analyses.

### Statistical Analyses

Statistical procedures were performed using SPSS for Windows version 16.0 (SPSS Inc, Chicago, IL). Statistical significance was defined to be p ≤ 0.05. Results with 0.05 < p < 0.10 were also noted to show close associations. Differences in the anthropometric and lipoprotein profiles in boys versus girls were performed using independent t tests. Dixon's test for outliers was used to identify unusual values. When identified, all data for that participant were excluded from further analyses. Using Dixon's test, data for 4 children were excluded. Data for the variables of interest were not significantly skewed. Correlations were used to evaluate bivariate associations among dependent and independent variables including intakes of total energy, fat, carbohydrate and protein, and biochemical and blood pressure variables of interest. As associations between these variables were similar for Pearsons and Spearmans correlations, values are reported only for Pearsons correlations, and parametric tests were used in subsequent analyses. Multiple linear regression analyses were used to assess the relationship of total energy intake (unadjusted for source of energy), or, individually, of fat, carbohydrate and protein to TC, VLDL-C, IDL-C, LDL-C, HDL-C, TG, HOMA-IR, NEFA, ISI-FFA, sBPz and dBPz after adjusting for gender, pubertal stage and waist circumference of the participating child. Child age was not included as a covariate since it was not significantly correlated with dependent or independent variables.

As might be expected BMIz and WC were highly correlated (r = 0.87, p < 0.001). In addition, WC and BMI were both associated with markers of cardio metabolic risk factors including TG (BMIz r = 0.34; WC r = 0.30), HDL-C (BMIz r = -0.40; WC r = -0.46), HOMA-IR (BMIz r = 0.60; WC r = 0.57), ISI-FFA (BMIz r = -0.57; WC r = -0.55), sBPz (BMIz r = 0.25; WC r = 0.28) dBPz (BMIz r = 0.23; WC r = 0.87). Because WC is included in the National Cholesterol Education Program (NCEP) ATP III recommendations [34], WC was chosen for results presentation and discussion.

## Results

Girls enrolled in this study were sexually more mature than boys and had significantly higher body weight, body fat %, BMI-z scores, WC, TG, insulin, HOMA-IR and NEFA than boys (Table 1). Boys had significantly higher ISI-FFA than girls. Intakes of energy and macronutrients did not differ significantly by gender.

Pearson correlations were used to evaluate the inter-relationship between selected cardio metabolic risk factors. Although the lipoprotein cholesterol subclasses tended to be inter-related, HDL-C was not significantly associated with LDL-C and IDL-C; and TG was not significantly associated with TC. LDL-C was significantly associated with IDL-C (r = 0.61, p < 0.001) and VLDL-C (r = 0.32, p < 0.01). TC was significantly associated with all lipoprotein cholesterol subclasses i.e. HDL-C (r = 0.41, p < 0.001), LDL-C (r = 0.94, p < 0.001), IDL-C (r = 0.60, p < 0.001) and VLDL-C (r = 0.29, p < 0.01). As expected, TG was highly associated with VLDL-C (r = 0.88, p < 0.001) and IDL-C (r = 0.64, p < 0.001). HOMA-IR and ISI-FFA were highly and negatively associated (r = -072, p < 0.001). HOMA-IR was also associated with HDL-C (r = -0.33, p < 0.001), VLDL-C (r = 0.29, p < 0.01) and TG levels (r = 0.48, p < 0.001) whereas ISI-FFA was significantly associated with HDL-C (r = 0.43, p < 0.001) and TG (r = -0.44, p < 0.001). sBPz was associated with VLDL-C (r = 0.23, p < 0.05) and dBPz (r = 0.37, p < 0.001). dBPz was significantly associated with LDL-C (r = 0.24, p < 0.05), IDL-C (r = 0.31, p < 0.05), VLDL-C (r = 0.39, p < 0.01), TG (r = 0.31, p < 0.01).

**Table 1:** Characteristics of participating African-American Boys and Girls

	**Boys (n = 36)**	**Girls (n = 44)**	***p*-value**^a^
	**Mean (SD)**		
**Anthropometrics**			
Age (years)	10.72 (1.06)	10.53 (1.12)	*ns*
Pubertal stage (1-5)	2.24 (1.52)	3.42 (1.21)	0.001
Height (m)	1.48 (0.09)	1.50 (0.08)	*ns*
Weight (kg)	57.62 (18.11)	66.34 (14.23)	0.010
Body fat (%)	32.34 (9.02)	40.63 (7.70)	<0.001
BMI-z score	1.82 (0.51)	2.13(0.46)	0.005
WC (cm)	83.62 (15.51)	90.73 (12.63)	0.020
**Biochemical parameters**			
TC (mmol/l)	4.23 (0.14)	4.43 (0.15)	*ns*
HDL-C (mmol/l)	1.48 (0.03)	1.38 (0.12)	*ns*
LDL-C (mmol/l)	2.34 (0.21)	2.62 (0.33)	*ns*
IDL-C (mmol/l)	0.21(0.08)	0.24 (0.21)	*ns*
VLDL-C (mmol/l)	0.39 (0.03)	0.41(0.01)	*ns*
TG (mmol/l)	0.68 (0.16)	0.83 (0.23)	0.020
Insulin (μIU/ml)	8.01 (4.91)	13.06 (5.08)	0.001
HOMA-IR	1.71 (1.12)	3.20 (2.79)	0.001
NFFA (mEq/L)	25.72 (5.22)	28.93 (4.54)	0.005
ISI-FFA	0.41 (0.21)	0.21 (0.14)	<0.001
**Blood Pressure**			
sBP (mm Hg)	105.94 (8.90)	104.32 (7.22)	*ns*
dBP (mm Hg)	61.73 (8.67)	62.25 (7.07)	*ns*
sBP-z score	0.03 (0.74)	-0.13 (0.75)	*ns*
dBP-z score	-0.06 (0.71)	-0.03 (0.67)	*ns*
**Dietary variables**			
Total energy (kcal/day)	1805.03 (534.42)	1743.95 (593.91)	*ns*
Carbohydrate (g/day)	210.41 (64.62)	222.74 (77.50)	*ns*
Fat (g/day)	77.94 (27.72)	69.24 (29.75)	*ns*
Protein (g/day)	68.84 (22.01)	62.85 (21.92)	*ns*

^a^*p *values calculated from two-sample *t*-tests

ns, non significant; WC, waist circumference; TC, total cholesterol; HDL-C, HDL cholesterol; LDL-C, LDL cholesterol; IDL-C, IDL cholesterol; VLDL-C, VLDL cholesterol; TG, triglycerides; HOMA-IR, homeostasis model assessment of insulin resistance; NEFA, Non-esterified fatty acids; ISI-FFA, fatty acid insulin sensitivity; sBP, systolic blood pressure; dBP, diastolic blood pressure.

Also, using bivariate analysis, total energy intake was significantly correlated with IDL-C (r = 0.22, p < 0.05), and VLDL-C (r = 0.22, p < 0.05) and sBP (r = 0.23, p < 0.05). Carbohydrate intake was positively associated with TG (r = 0.27, p < 0.05), VLDL-C (r = 0.30, p < 0.01) and IDL- C (r = 0.34, p < 0.01). Protein and Fat intake were associated by sBP (r = 0.22, p < 0.05 for both).

Using regression analysis, total energy intakes, without adjustment for the source of the energy, were significantly and positively associated with IDL-C (p < 0.05) and VLDL-C (p < 0.05) after accounting for child's gender, pubertal stage and WC (Model 1, Table 2).

**Table 2:** Standardized regression coefficients from multiple linear regression models evaluating the relationship between total energy and individual macronutrients intakes, and cardio metabolic risk factors (n = 80).

**Dependent variables**	**Independent Variables**			
	**Model 1**^a^	**Model 2**^a^		
	**Total Energy**	**Carbohydrate**	**Fat**	**Protein**
**TC**	0.162	0.053	-0.129	0.288
**HDL-C**	-0.030	-0.035	-0.035	0.044
**LDL-C**	0.171	0.031	-0.107	0.300
**IDL-C**	0.243*	0.457**	-0.437*	0.276
**VLDL-C**	0.232*	0.396**	-0.206	0.068
**TG**	0.171	0.422**	-0.163	-0.085
**HOMA-IR**	-0.024	0.302*	-0.025	-0.341*
**NEFA**	-0.141	0.077	-0.127	-0.116
**ISI-FFA**	0.052	-0.206#	0.138	0.139
**sBPz**	0.208#	0.119	0.108	0.001
**dBPz**	0.136	0.218	-0.085	0.020

Significance: # for 0.05<p < 0.10, * for p < 0.05, ** for p < 0.01, *** for p ≤ 0.001.

^a^Multiple linear regression models for total energy (Model 1-- no adjustment for source of energy) and macronutrients (Model 2-- influence of one change in one macronutrient assessed when intakes of the other 2 macronutrients are held constant) after adjusting for gender, pubertal stage and WC (independent variables).

TC, total cholesterol; HDL-C, HDL cholesterol; LDL-C, LDL cholesterol; IDL-C, IDL cholesterol; VLDL-C, VLDL cholesterol; TG, triglycerides; HOMA-IR, homeostasis model assessment of insulin resistance; NEFA, Non-esterified fatty acids; ISI-FFA, fatty acid insulin sensitivity; sBP, systolic blood pressure; dBP, diastolic blood pressure.

In a second regression model (Model 2, Table 2), carbohydrate intakes, with fat and protein intakes held constant, were significantly and positively associated with IDL-C (p < 0.01), VLDL-C (p < 0.01), TG (p < 0.01) and HOMA-IR (p < 0.05); and closely but negatively associated with ISI-FFA (0.05 < p < 0.10). Fat intakes, with carbohydrate and protein intakes held constant, were found to be significantly and negatively associated with IDL-C (p < 0.05). Protein intakes, with fat and carbohydrate held constant, was negatively associated with HOMA-IR (p < 0.05).

**Figure 1:**
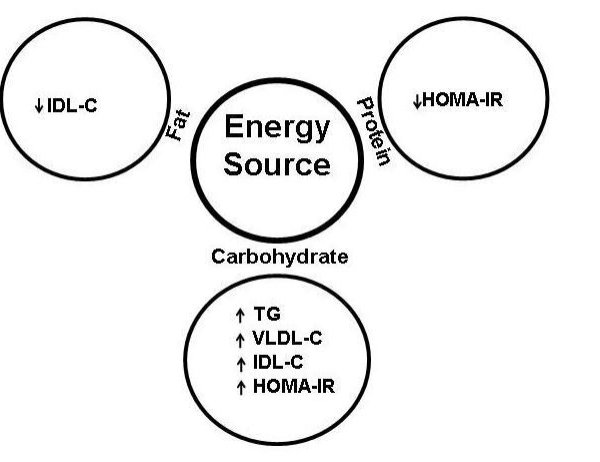
**Association between source of energy and cardio metabolic risk factors**. HOMA-IR, homeostasis model assessment of insulin resistance; IDL-C, IDL cholesterol; V LDL-C, VLDL cholesterol; TG, triglycerides; ISI-FFA, fatty acid insulin sensitivity.

Figure 1 summarizes the association between macronutrients and selected cardio metabolic risk factors. Increases in energy from carbohydrate were associated with the undesirable effect of increased HOMA-IR, TG, VLDL-C and IDL-C. Increases in protein energy were associated with the desirable effect of reduced HOMA-IR; and increase in fat energy was associated with the desirable effect of reduced IDL-C. The significance of results from multivariate regression analyses were unchanged regardless of whether BMIz or WC was included in the models (data with BMIz not presented). Additionally, the significance of results were unchanged regardless of whether child gender or pubertal stages were included in the models (data without gender and pubertal stage not presented).

## Discussion

Our main outcomes highlight the association between intakes of energy from each of the three classes of macronutrient and important cardio metabolic risk factors in high BMI, AA children. Although the risk for type2 diabetes is known to be higher for AA than white children [35], few data on the influence of specific macronutrients on risks for this condition in AA children are available. We (20) and others [36,37] have previously reported that obesity in AA children predisposes them to insulin resistance early in life. In the present analyses, WC and BMI z-scores both showed positive associations with TG, HOMA-IR, sBPz, dBPz and negative association with HDL-C and ISI-FFA. This is also consistent with previous studies reporting that overweight status and obesity are important risk factors CVD [3,4,6,7]. WC, used in these analyses, is a better assessment of visceral adiposity than BMI or skin-fold measurements [38], and is one of the criteria used to study cardio metabolic risk [39].

In Model 1 (Table 2), increases in energy intakes (regardless of source) were associated with increased VLDL-C and IDL-C but not significantly with any of the other cardio metabolic risk factors evaluated in this study. A larger sample size would be needed to determine whether the close association (0.05 < p < 0.10) with sPBz would then be significant or not. Evaluating the influence of the type of macronutrient contributing in the increased energy, however, provides additional insight. To do this, all 3 macronutrients were entered simultaneously into a second regression model, allowing the influence of one to be evaluated with levels of the other two held constant. This approach differs from approaches often used by others to evaluate the relationship of macronutrients to cardio metabolic risk factors [10,15].

In Model 2, with fat and protein intakes fixed, increased intakes of energy from carbohydrate were associated with undesirable increases in circulating levels of IDL-C, VLDL-C, TG and hepatic insulin resistance (HOMA-IR). Carbohydrate also showed a close negative association (0.05 < p < 0.10) with adipose tissue insulin sensitivity (ISI-FFA). It is likely that increased carbohydrate energy intake affects the adipocyte by suppressing the efficiency with which insulin exerts its inhibitory effect on TG hydrolysis, allowing NEFA release from endogenous TG. Accumulation of NEFA in plasma indicates energy-replete peripheral cells, promoting hepatic incorporation of these fatty acids, along with newly synthesized NEFA, into TG which will be packaged into VLDL and metabolized to IDL. Unfavorable relationships between higher carbohydrate and TG [10,13,40] and the prevalence of atherogenic dyslipidemia [41,42] have been observed by others also. Independently of TG, however, high NEFA levels are known to be strongly associated with multiple indices of metabolic dysfunction including metabolic syndrome and cardiovascular mortality [43]. In our study, although carbohydrate intakes were not associated with increased concentrations of NEFA, the close and negative association between carbohydrate intake and ISI-FFA (0.05 < p < 0.10) and the significant association with HOMA-IR (p < 0.05) suggests that carbohydrate may negatively affect insulin sensitivity in both hepatocytes and adipocytes.

With carbohydrate and fat intakes held constant, increased intakes of energy from protein were not significantly associated with levels of circulating lipids or lipoproteins, although there was a significant protective effect of protein on hepatic insulin sensitivity (HOMA-IR). Others have reported that increasing percent energy from protein (without adjusting for energy from carbohydrate or fat) is associated with significantly lower fasting glucose concentrations in AA children [15]. It is possible that the effects ascribed to protein by others are actually due to reciprocal decreases in one or both of the other macronutrients.

In Model 2 (Table 2), with carbohydrate and protein intakes held constant, increased intakes of energy from fat was associated with significantly lower IDL-C. A significant negative relationship between TG concentrations and fat intake has been reported previously in AA children [15] but the effect of fat in that study could not be distinguished from corresponding changes in carbohydrate and/or protein. In our study, after controlling both carbohydrate and protein, fat intake was not significantly associated with TG concentrations in AA children.

Although high BP is associated with obesity in AA children [44], a lack of relationships between BP and dietary energy has been reported previously [15,16]. In our study, neither total energy intake, nor intakes of energy from any of the three macronutrients was significantly associated with either systolic or diastolic blood pressures in these children.

Limitations of this study include restriction to low-income, inner-city, African American children and exclusion of children with BMI's less than the 85^th ^percentile when matched for age and gender. These limitations preclude comparisons among children of different races, ages and socioeconomic backgrounds, and comparisons with lower BMI children. The limitations inherent in collecting dietary data, regardless of population, are also recognized. This is a cross-sectional analysis of data, precluding a cause and effect relationship. Future longitudinal studies with measurements at several time-points would be needed to evaluate a causal relationship.

## Conclusion

Based on the data in this study, we conclude that increases in energy from carbohydrate should be discouraged, whereas increases from protein or fat would be of less concern. Associations in this study, between diet components and cardio metabolic risk factors point to the need to target these children for nutrition intervention.

## Abbreviations

AA: African American; BMI: Body mass index; CVD: Cardio vascular disease; dBPz: diastolic blood pressure z-score; HDL-C: High density lipoprotein cholesterol; HOMA-IR: Homeostasis model assessment of insulin resistance; IDL-C: Intermediate density lipoprotein cholesterol; ISI-FFA: Fatty acid insulin sensitivity; LDL-C: Low density lipoprotein cholesterol; NEFA: Non-esterified fatty acids; sBPz: Systolic blood pressure z-score; TC: Total cholesterol; TG: Triglyceride; VLDL-C: Very low density lipoprotein cholesterol; WC: Waist circumference.

## Conflict of interests

The authors declare that they have no competing interests.

## Authors' contributions

Contributor's list: SS contributed in statistical analysis, prepared the manuscript and submission. LSR participated in the development of the protocol, analytical framework for the study and patient screening. MLH provided statistical expertise. RHL provided expertise as a pediatric endocrinologist and child health specialist. SEF was the principal investigator of the study. She supervised the design and execution of the study and manuscript.
